# Glucosyltransferase CsUGT78A14 Regulates Flavonols Accumulation and Reactive Oxygen Species Scavenging in Response to Cold Stress in *Camellia sinensis*

**DOI:** 10.3389/fpls.2019.01675

**Published:** 2019-12-27

**Authors:** Mingyue Zhao, Jieyang Jin, Ting Gao, Na Zhang, Tingting Jing, Jingming Wang, Qiuyan Ban, Wilfried Schwab, Chuankui Song

**Affiliations:** ^1^State Key Laboratory of Tea Plant Biology and Utilization, International Joint Laboratory on Tea Chemistry and Health Effects, Anhui Agricultural University, Hefei, China; ^2^Biotechnology of Natural Products, Technische Universität München, Freising, Germany

**Keywords:** *Camellia sinensis*, tea plant, glucosyltransferase, reactive oxygen species scavenging capacity, flavonoids, cold tolerance

## Abstract

Glycosyltransferases (UGTs) play diverse roles in cellular metabolism by altering regulatory metabolites activities. However, the physiological roles of most members of UGTs in crops in response to abiotic stresses are unknown. We have identified a novel glycosyltransferase CsUGT78A14 in tea crops, an important economic crops, whose expression is strongly induced by cold stress. Biochemical analyses confirmed that CsUGT78A14-1 showed the highest activity toward kaempferol and is involved in the biosynthesis of kaempferol-diglucoside, whereas the product of CsUGT78A14-2, which differs from CsUGT78A14-1 by a single amino acid, was identified as 3-O-glucoside. The accumulation of kaempferol monoglucosides and diglucosides was consistent with the expression levels of CsUGT78A14 in response to cold stress, as well as in different tissues and genotypes of tea plants. Down-regulation of *CsUGT78A14* resulted in reduced accumulation of flavonols, reactive oxygen species (ROS) scavenging capacity and finally reduced tea plant stress tolerance under cold stress. The antioxidant capacity of flavonols aglycon was enhanced by glucosylation catalyzed by CsUGT78A14. The results demonstrate that CsUGT78A14 plays a critical role in cold stress by increasing flavonols accumulation and ROS scavenging capacity, providing novel insights into the biological role of UGTs and flavonoids in plants.

## Introduction

Low temperatures, including chilling and/or freezing temperatures, are one of the most important environmental factors that adversely affect plant growth and agricultural productivity (Chinnusamy et al., 2007). Some plants can enhance their freezing tolerance after exposure to low but non-freezing temperatures for a period of time (Thomashow, 1999). As one of the most important economic crops, tea plant (*Camellia sinensis*) is widely cultivated in almost 30 countries (Chen et al., 2007). Although tea plant can be grown in various regions, low temperatures are still one of the most important factors that limit its distribution mainly in tropical and subtropical climates because of the thermophilic nature of tea plants (Wang et al., 2012). Therefore, it is essential to understand the physiological response of tea plants exposed in cold stress and explore approaches to improve tea plants cold tolerance.

Plant growth and development can be severely effected by environmental stresses, including cold stress. Exposure of plants to low temperatures can cause an accumulation of reactive oxygen species (ROS) (Manthey et al., 2009), which are recognized as a common risk from abiotic stress and highly reactive and toxic, and affect many cellular functions (Gill and Tuteja, 2010). A series of protective mechanisms are triggered when plants sense the cold temperature (Iba, 2002; Wang et al., 2013). It is well known that the induction of the antioxidant capacity for ROS scavenging is important to protect plants in stresses, including cold stress (Ning et al., 2010; You et al., 2014; Hazman et al., 2015; Li et al., 2017). The accumulation of flavonoids can be induced by biotic and abiotic environmental stresses in plants (Dixon and Paiva, 1995). Flavonoids are among the most bioactive plant secondary metabolites, and are regarded as no enzymatic defense components because of their ROS scavenging capacity (Bolwer et al., 1992). Therefore, flavonoids are important for plants to protect themselves under environmental stresses (Winkel-Shirley, 2002). Over-accumulation of antioxidant flavonoids leads to an enhancement of drought tolerance in *Arabidopsis* (Nakabayashi et al., 2014). However, the biological role of flavonoid glycosides is not clear as they are considered to be less effective anti-oxidants in comparison with the corresponding aglycones (Vogt and Jones, 2000; Gachon et al., 2005). Therefore, the precise role(s) of glycosylation in abiotic stresses is still very difficult to understand until now.

In tea plant, flavonoids are the predominant secondary metabolites, mainly in the form of glycosides. Glycosylation, mediated by diphosphate-dependent glycosyltransferases (UGTs), renders the flavonoids more water soluble and less toxic, and also enable their transport (Bowles et al., 2006; Song et al., 2018). Recently, 132 UGTs were identified in a transcriptome database of the tea plant. The function of most of them were unknown, except four UGTs (CsUGT78A14, CsUGT78A15, and CsUGT82A22, CsUGT73A20) which exhibited catalytic activity toward phenolic acids and flavonoid (Cui et al., 2016; Zhao et al., 2017), and three UGTs involved in the glycosylation of aroma (Ohgami et al., 2015; Jing et al., 2019; Song et al., 2018). Numerous studies have indicated that some UGTs are involved in the regulation of plant growth and development in responses to biotic and abiotic stresses (Tognetti et al., 2010; von Saint Paul et al., 2011; Liu et al., 2015; Palareti et al., 2016), and improved freezing tolerance in *Arabidopsis* (Schulz et al., 2016). However, little is known about the physiological roles of most members of the plant UGTs. The response mechanisms of plants to environmental changes and how these glycoconjugates can contribute to plant protection is still a major challenge.

In this study, the *C. sinensis* UGT gene *UGT78A14* was identified as a gene involved in the regulation of plant cold stress tolerance. The expression of *UGT78A14* was strongly induced by cold stress. *In vitro* assays showed that CsUGT78A14-1 and -2 could catalyze the glucosylation of flavonols and the main products were identified as kaempferol-diglucoside, and 3-O-glucoside, respectively. Down-regulation of *UGT78A14* in the tea plant resulted in reduced accumulation of flavonol glycosides, ROS scavenging capacity and finally reduced tea plant cold stress tolerance. The antioxidant and ROS scavenging capacity of the flavonols was greatly enhanced by glucosylation catalyzed by UGT78A14-1 compared to the corresponding free aglycons. These results provide novel insights into the biological role of flavonoid glycosides in plants, and deepen our knowledge of the response mechanisms of flavonol glycoconjugates to stress in plants.

## Materials and Methods

### Plant Materials

Tea plant samples were collected from the tea plant cultivar and Germplasm Resource Garden of Anhui Agricultural University (Guohe Town) and were immediately frozen in liquid nitrogen. The tea plant samples from *C. sinensis* var. *sinensis* cv. ‘Shuchazao,’ ‘Mingxuan213,’ ‘Zhenghedabai,’ ‘Longjingchangye,’ ‘Mingshanbaihao,’ ‘Yingshuang,’ ‘Longjing43,’ and ‘Fuzao2’ were used for metabolites and transcripts analyses. All samples were stored at −80°C until use.

### Chemicals and Reagents

All biochemicals including kaempferol, quercetin, myricetin, uridine diphosphate glucose (UDP)-glucose, UDP-galactose, and UDP-glucuronic acid were purchased from Sigma (St. Louis, MO, USA). All other chemicals and solvents were obtained from Sigma or Aladdin (Shanghai, China), unless otherwise noted.

### Cold Stress Treatment

For metabolites and transcripts analysis, 1-year-old tea plants were grown at 80% relative humidity and 16 h/8 h light/dark condition. The tea plants were first treated at 4°C, and leaves were collected after 6 h short-term cold stimulus (CS) and 7 days long-term chilling acclimation (CA). Then, the plants were transferred to 0°C for an additional 7 days long-term freezing acclimation (FA). After that, the plants were moved to control conditions (25°C) for 7 days long-term de-acclimation (DA) according to a previous study (Li et al., 2019). The control plants and *UGT78A14*-silenced tea plants were exposed to −5°C for 3 h for cold stress treatment. At least three experimental replicates were conducted for the treatment and control. Young tissues with one bud and two leaves were harvest and were used to determine of antioxidant activity and ROS content.

### Ribonucleic Acid Isolation and Complementary Deoxyribonucleic Acid Synthesis

Total RNA from leaves of Shuchazao was isolated using RNAiso-matefor Plant Tissue (Takara, Dalian, China) and RNAiso Plus (Takara, Dalian, China). The cDNA was synthesized by reverse transcription from total RNA using PrimeScriptRT Master Mix (Takara, Dalian, China).

### Quantitative Real-Time Polymerase Chain Reaction Analysis

Real-time PCR was performed according to our published protocols (Song et al., 2015b; Jing et al., 2018) with gene-specific primers (**Table S1**). The glyceraldehyde-3-phosphate dehydrogenase (GAPDH) gene was used as an internal reference gene. The relative expression was calculated using the 2^–ΔΔCT^ method (Livak and Schmittgen, 2001). All qRT-PCR in this study were performed in three biological replicates, and each of which consisted of three technical replicates.

### Expression Vector pGEX-4T1-UGTs

The full-length *UGT*s were amplified by PCR from the cDNA of tea plants leaves (primers as shown in **Table S1**). The full-length coding sequences were amplified using proofreading Phusion DNA polymerase according to a published protocol (Jing et al., 2019). The amplified full-length sequences were digested with BamHI and SalI, the resulting gene fragments were cloned into pGEX-4T1 vector, and the recombinant plasmids were subsequently transformed into Trans1T1-competent cells.

### Heterologous Protein Expression and Purification

Expression constructs harboring the pGEX-4T1-UGTs and control plasmids were all transformed into *Escherichia coli* strain BL21 (DE3) pLysS cells (Jing et al., 2019). Protein expression was induced by adding 1 mM (final concentration) isopropyl-ß-D-thio-galactopyranoside. The culture was incubated at 16°C overnight. The next day, the proteins were purified by GST bind resin (Jing et al., 2019). Protein concentration was determined by a photometric method (Bradford, 1976).

### Enzymatic Activity Assay

Each reaction mixture (5 μl in total) contained 50 mM Tris-HCl buffer (pH 7.5, 10% glycerol, and 10 mM 2-mercaptoethanol), 250 mM UDP-glucose, alcohol substrates, and purified protein (0.5–1 μg per reaction) was used for the initial screening according to Jing et al., (2019) with some motifications. The reaction mixture was incubated for 30 min at 30°C, the reaction was stopped by adding reaction solution of UDP-Glo™ assay reagent (Sheikh et al., 2017). Three biological replicates were carried out. The best reaction temperature and pH was tested according to Jing et al., (2019). For the determination of the kinetic parameters of CsUGT78A14, the sugar donor was fixed at 100 μM, and at least seven different substrate concentrations covering the range from 1 to 500 μM were used at the optimized conditions.

### Identification of Products by Liquid Chromatography–Mass Spectrometry

Reaction mixtures contained 5 mM UDP-glucose, 200 μM substrate, and the purified protein (1 to 2 μg) were incubated at 30°C for 2–3 h, the reaction were extracted with 200 μl ethyl acetate for two times. Ethyl acetate was vaporized and the residue was dissolved in 50 μl methanol/water (1:1, v/v) for products identification by liquid chromatography–mass spectrometry (LC–MS) (Jing et al., 2019). Products were identified by comparison of their retention time and MS spectra with those from literature or reference material.

### Gene Suppression of *CsUGT78A14* in Tea Using Candidate Antisense Oligonucleotides

Candidate antisense oligonucleotides (AsODN) with complementarity to the segment of the target gene were selected using Soligo software (Ding and Lawrence, 2003) with *CsUGT78A14* as input sequence (**Table S1**). AsODNs were synthesized by General Biosystems Company. To silence the genes, tea leaves were grown in Eppendorf tubes (2 ml) containing 40 μM AsODN-CsUGT78A14 solution for 24 h, the sense oligonucleotides (sODN) were used as control. To silence CsUGT78A14 in the tea leaves attached to the whole tea plant, 1 ml 20 μM AsODN-CsUGT78A14 solution were injected into the tea seeding. Five experimental replicates were conducted for the treatment and control. After 12, 24, and 48 h incubation, the leaves were exposed to −5°C for 3 h, and then harvested and kept at −80°C prior to analysis (Liu et al., 2018).

### Metabolite Analysis

The materials collected in this study were ground and kept at −80°C prior to analysis. For metabolites analysis, 50 mg samples were extracted with 1 ml 75% (v/v) methanol for two time; 3 μg ml^−1^ chlorophenylalanine solution was added as internal standard. The metabolites were extracted and sonicated for 20 min at 4°C. After that, the mixture was centrifuged at 12,000 rpm for 10 min under 4°C. The supernatants were used for glycoside analysis by LC–MS. Five experimental replicates were conducted metabolites analysis.

### Determination of Fv/Fm

Twenty four hours after the CsUGT78A14 was silenced in tea in the seeding or tea leaves using AsODNs, and tea leaves or seedings were kept in −5°C temperature for 3 h and then recover for 3 h in 25°C conditions. Net photosynthetic rate and maximum photochemical efficiency and of PSII (Fv/Fm) were measured (Li et al., 2018c). Tea plants without cold treatment were used as controls. All Fv/Fm determination were performed at least in three biological replicates, and each of which consisted of five technical replicates.

### Diaminobenzidine and Nitrobluetetrazolium Staining

The superoxide radical and H2O2 were detected using histochemical staining with nitrotetrazolium blue chloride (NBT) and diaminobenzidine (DAB) as described (Romero-puertas et al., 2004). For both staining methods, the seedlings were decolorized in 90% ethanol, followed by 100% ethanol, and then take photographs (Li et al., 2018a). Three biological replicates were carried out.

### Determination of Antioxidant Activity and Reactive Oxygen Species Content

The total antioxidant activity was determined using the ferric reducing ability of plasma (FRAP) method with a FRAP reagent kit (Beyotime, Shanghai, China), 2,2’-azino-bis(3-ethylbenzothiazoline-6-sulfonic acid) (ABTS) method (Beyotime, Shanghai, China), and 2,2-diphenyl-1-picrylhydrazyl (DPPH) as described previously (Tohge et al., 2005b). At least three biological replicates were carried out.

## Results

### Expression Patterns of *CsUGT78A14* During Cold Acclimation and Deacclimation

**Figure 1:**
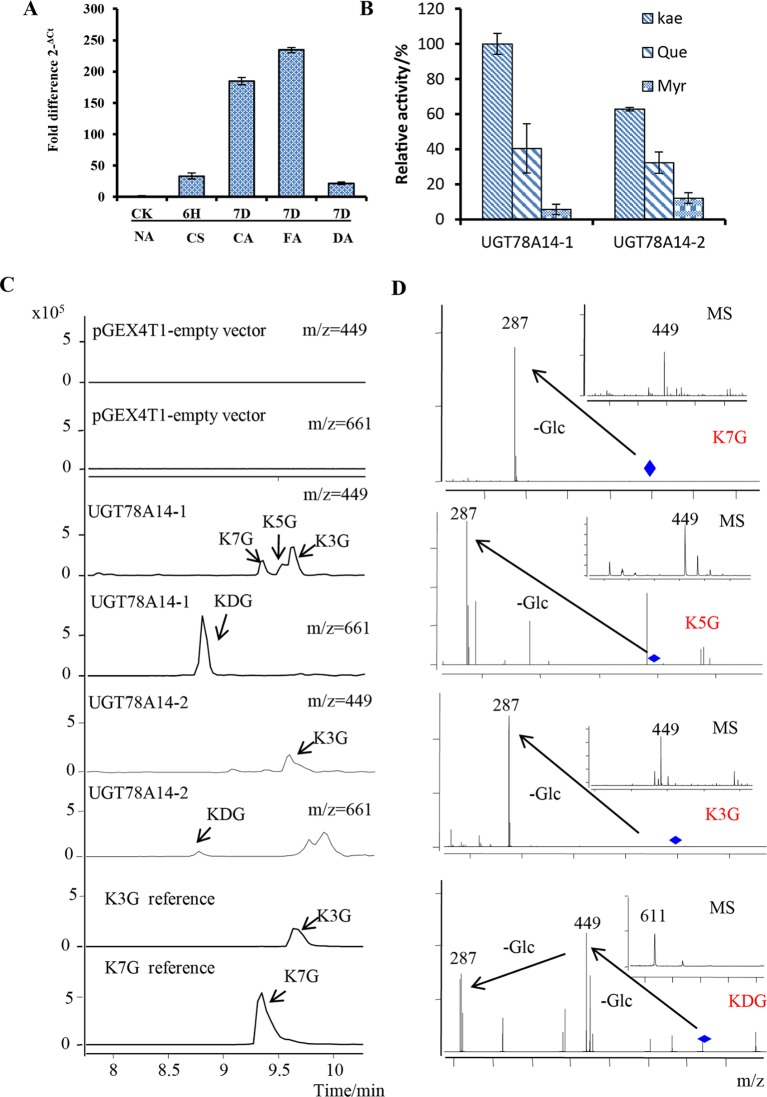
*CsUGT78A14* expression during cold stress and enzymatic analysis of its encoded protein. Expression patterns of CsUGT78A14 during cold acclimation and de-acclimation **(A)**. Enzymatic analysis of the recombinant UGT proteins encoded by CsUGT78A14-1, and CsUGT78A14-2 with kaempferol, quercetin, and myricetin as substrates **(B)**. Liquid chromatography–mass spectrometry analysis of enzymatically formed products by CsUGT78A14-1, CsUGT78A14-2, and empty vector control **(C)**. Mass spectra of formed products by CsUGT78A14-1, CsUGT78A14-2 **(D)**. Data are presented as mean ± SE of at least three biological replicates.NA: tea plants were grown at room temperature. CS, cold stimulus, tea plants were grown at 4°C for 6 h. CA, chilling acclimation, tea plants were grown at 4°C for 7 days. FA, freezing acclimation, tea plants were first treated at 4°C for 7 days, and then treated at 0°C for an additional 7 days. DA, de-acclimation, tea plants were first treated at 4°C for 7 days, then at 0°C for additional 7 days, and moved to normal condition 25°C for 7 days. K3G, kaempferol-3-O-glucoside; K5G, kaempferol-5-O-glucoside; K7G, kaempferol-7-O-glucoside; KDG, kaempferol-diglucoside.

Multi-omics data deposited on the Tea Plant Information Archive (TPIA) showed that TEA007509 was strongly responsive to low temperatures (Li et al., 2019 and Wang et al., 2013). To validate this, 1-year old tea plants were grown under the cold stress condition and transcript levels were measured by real-time qRT-PCR (**Figure 1A**). When the plant were treated with cold stress, the expression levels of TEA007509 in the leaves were increased by approximately 40-fold and 180-fold after 6 h (CS) and 7 days (CA), respectively, when compared with the control (**Figure 1A**). The expression levels continue induced when plants were transferred to 7-day FA treatment at 0°C. It should be noted that the mRNA expression level of TEA007509 was significantly down-regulated when the plants was de-acclimated for 7 days (**Figure 1A**). The gene was assigned to CsUGT78A14 by the UGT Nomenclature Committee (Mackenzie et al., 1997). This observation suggest that CsUGT78A14 clearly responded to cold stress and thus investigated its relevance in cold stress resistance of tea plant.

### UGT78A14-1/-2 Specifically Catalyze the Glucosylation of Kaempferol

To verify the function of CsUGT78A14, we first characterized the enzymatic activities of its encoded proteins. Two alleles of CsUGT78A14 (assigned to CsUGT78A14-1 and -2) were obtained from *C. sinensis* var. *sinensis* cv. Shuchazao. These UGTs were expressed in *E. coli* BL21 and the purified protein was verified by SDS–PAGE (**Figure S1**).

The enzymatic activities of these two proteins were tested with kaempferol, quercetin, and myricetin as selected substrates. Both CsUGT78A14-1 and -2 could use kaempferol and quercetin as a substrate (**Figure 1B**) when UDP-glucose was used as donor substrate. The enzymatic activities of CsUGT78A14-1 and -2 were further tested with 53 substrates selected from a wide range of chemical classes (**Figure S3**) and three donor sugars (**Figure S4**). Both CsUGT78A14-1 and CsUGT78A14-2 showed a similar substrate tolerance and preferred kaempferol and UDP-glucose as acceptor and donor substrates, respectively (**Figure 1B**). Low but detectable activity was measured toward quercetin (40% of that of kaempferol). However, the activity toward plant metabolites such as, myricetin, vanillic acid, vanillin, ferulic acid, gallic acid was negligible (**Figure S3**).

### A Diglucoside is the Main Product of UGT78A14-1 but -2 Only Forms Monoglucosides

The formation of glycosides was confirmed by LC-MS of enzyme assays containing UDP-glucose, acceptor substrate, and purified CsUGT78A14 protein. Interestingly, at least two product peaks were detected when using kaempferol and quercetin as acceptor substrates, indicated that CsUGT78A14 performed multiposition glycosylation on flavonols. The above-mentioned products were identified by retention time, parent ions (H adducts), and daughter ion spectra (MS/MS) in comparison with standards (Zhao et al., 2017). The main products of CsUGT78A14-1 was identified as a diglucoside by comparison of its parent and daughter ion spectra with a reference compound (**Figure 1D**) (Zhao et al., 2017), in addition to two monoglucoside products, identified as kaempferol-3-glucoside, and -7-glucoside (**Figure 1C**), when kaempferol was used as substrate. However, CsUGT78A14-2 only formed a trace amount of the diglucoside (**Figure 1C**) and one monoglucoside, which was identified as kaempferol-3-glucoside. Glucosides formed by CsUGT78A14-1 and -2 from quercetin was lower but the product profiles were identical to that of kaempferol (**Figure S5**). The kinetic parameters of recombinant CsUGT78A14-1 and CsUGT78A14-2 were also compared (**Figures S6**–**S8** and **Table 1**). These data indicated that the main product of UGT78A14-1 was a diglucoside, whereas the major product of UGT78A14-2, which differs from UGT78A14-1 in a single amino acid, was identified as 3-O-glucoside.

**Table 1:** Kinetic parameters of recombinant CsUGT78A14-1 and CsUGT78A14-2 proteins.

	Substrate	Km μM	Vmax nKat·mg^−1^	Kcat/K_M_ s^−1^ mM^-1^
**UGT78A14-1**	Kaempferol			
	Gal	27.1603	0.2155	0.411556
	Glc	27.0785	0.389	0.745146
	GA	47.7616	0.11933	0.129595
	Quercetin			
	Gal	4.9994	0.2008	2.083349
	Glc	9.3001	0.2917	1.626916
	GA	76.5363	1.0417	0.705978
**CsUGT78A14-2**	Kaempferol			
	Gal	64.5678	0.8616	0.692159
	Glc	8.1793	0.2369	1.502329
	GA	7.0651	0.2152	1.579939
	Quercetin			
	Gal	102.0854	6.4588	3.281742
	Glc	6.0176	0.2188	1.885994
	GA	6.0787	0.2229	1.902022

### Accumulation of Flavonol Glycosides and *UGT78A14* Transcripts in Different Tissues, Genotypes, and in Response to Cold Stress

**Figure 2:**
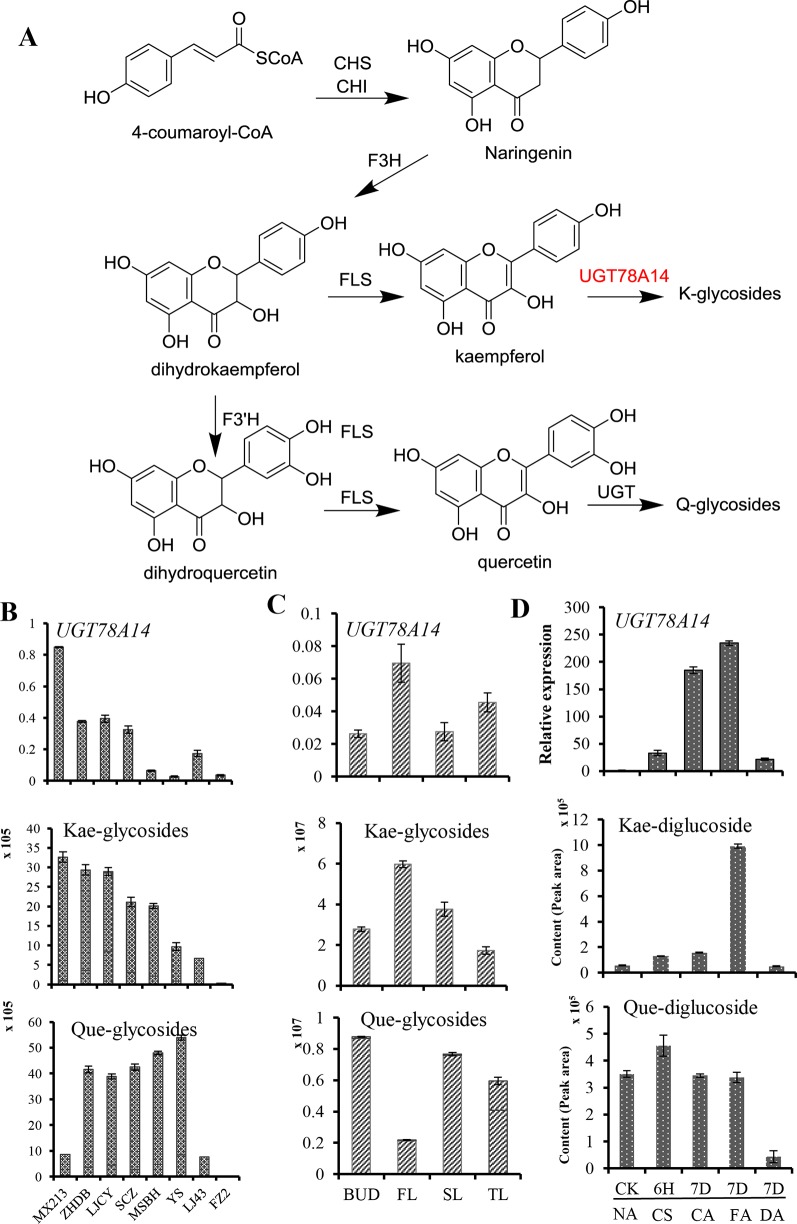
Flavonol glycosides accumulation and CsUGT78A14 transcripts in response to cold stress, and in different tissues and genotypes of tea plant.The biosynthesis pathway of kaempferol and quercetin glycosides in the tea plant **(A)**. Relative expression levels of CsUGT78A14 and accumulation of flavonoid glycosides in response to cold stress **(B)** in three different genotypes of tea plant **(C)**, as well as in different leaves (FL first; SL second; TL third) and one bud of the tea plant **(D)**. For cold stress conditions please refer to **Figure 1**. Data are presented as mean ± SE of at least three biological replicates.

We also assessed the relative levels of kaempferol and quercetin glycosides in leaves of eight different genotypes of the tea plant. Kaempferol glycosides were more abundant in *C. sinensis* var. *sinensis* cv. ‘Mingxuan213’ (MX213), cv. ‘Zhenghedabai’ (ZHDB), and cv. ‘Longjingchangye’ (LJCY) than in cv. ‘Yingshaung’ (YS) and cv. ‘Fuzao2’ (FZ2) (**Figure 2B**), consistent with the *UGT78A14* transcript levels in these genotypes. The relatively higher abundance of kaempferol glycosides and *UGT78A14* transcripts in different tea plant genotypes agreed with the UGT products observed in the *in vitro* assays. Further, consistent with the higher abundance of kaempferol in the first leaves, *UGT78A14* transcripts were also more abundant in the first leaves (**Figure 2C**). In contract, first leaves showed a very low content of quercetin glycosides. This analysis also indicated that kaempferol is more likely an *in planta* substrate of UGT78A14 than quercetin.

It should be noted that the amount of a putative kaempferol diglucoside (*m/z* 611 in positive mode) increased gradually at 4°C, and was strongly induced by five-fold when plants were transferred to 0°C for 7 more days. The concentration of this putative kaempferol diglucoside was lowered to the level of control when the plant was brought to room temperature for 7 days (**Figure 2D**). The correlation of *CsUGT78A14* transcript accumulation with the accumulation of flavonol glycosides, especially kaempferol glycosides (**Figure 2D**), showed that CsUGT78A14 plays a role in the production of the flavonol glycosides in response to cold stress in tea plant.

### Suppression of *UGT78A14* Reduces Flavonols Accumulation in Tea Plant

**Figure 3:**
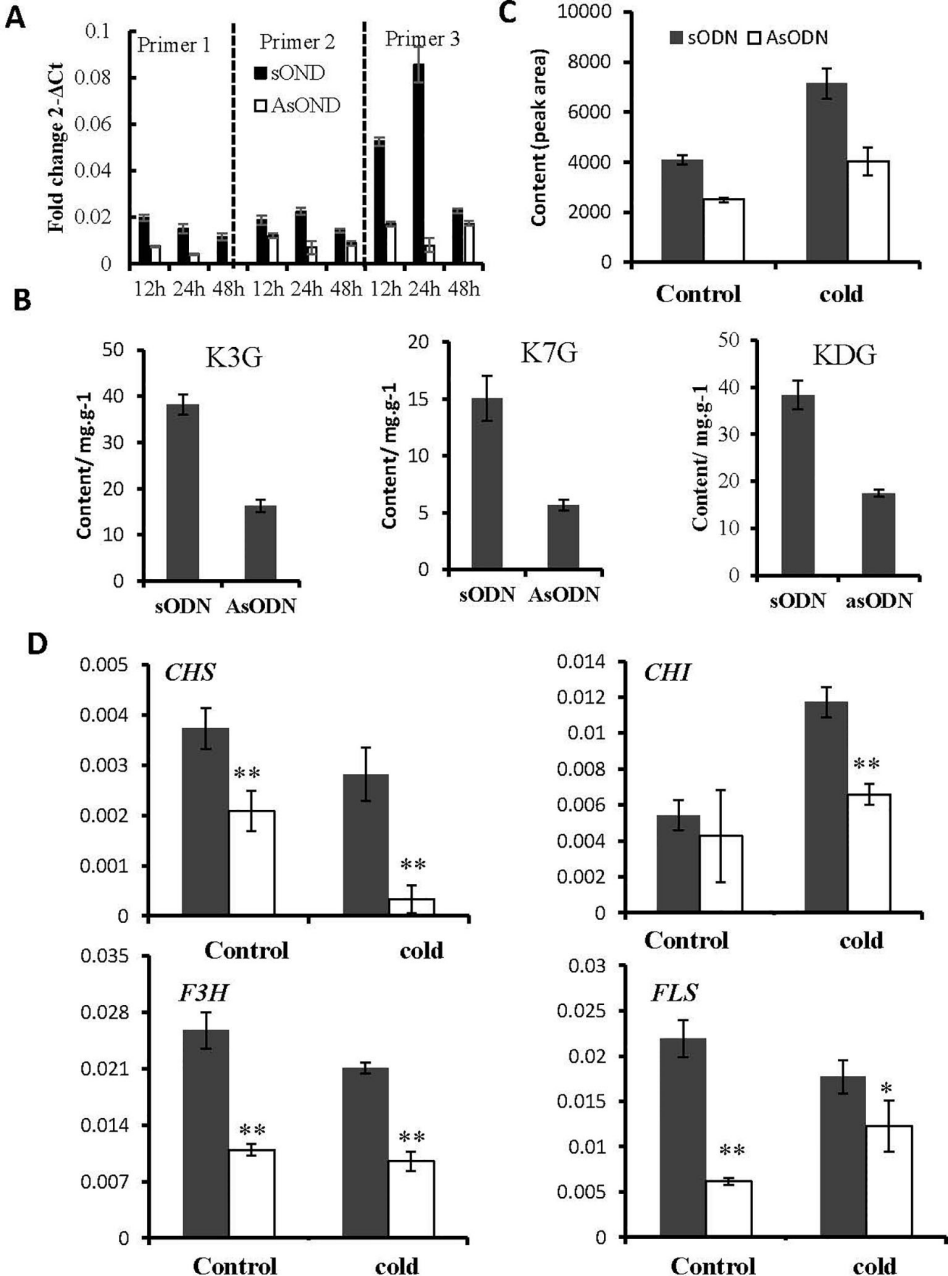
Suppression of CsUGT78A14 reduces flavonols accumulation and flavonoid-related genes expression in tea plant under normal and cold stress conditions. Quantitative PCR analysis of CsUGT78A14 expression in the control and *CsUGT78A14*-silenced tea leaves **(A)**. Liquid chromatography–mass spectrometry analyses of kaempferol glucosides in the control and *CsUGT78A14*–silenced tea leaves **(B)**. Flavonoid accumulation **(C)** and flavonoid-related genes expression **(D)** in the control and *CsUGT78A14*–silenced tea leaves. For all extractions and evaluations, at least three biological replicates were performed. Asterisks indicate significant differences relative to the control (Student’s t-test:*P < 0.05; **P < 0.01).

To obtain insight into the physiological roles of CsUGT78A14 in the tea plant, the expression level of *CsUGT78A14* was transiently suppressed in *C. sinensis* leaves by gene-specific antisense oligodeoxynucleotide suppression according to Zhao et al. (2019). Tea plants treated with sense oligodeoxynucleotide were used as control. The expression level of *CsUGT78A14* in tea leaves treated with AsODN_CsUGT78A14 was significantly reduced compared with the control (**Figure 3A**), which indicated that the AsODN method is effective for this gene in the tea plant.

**Figure 4:**
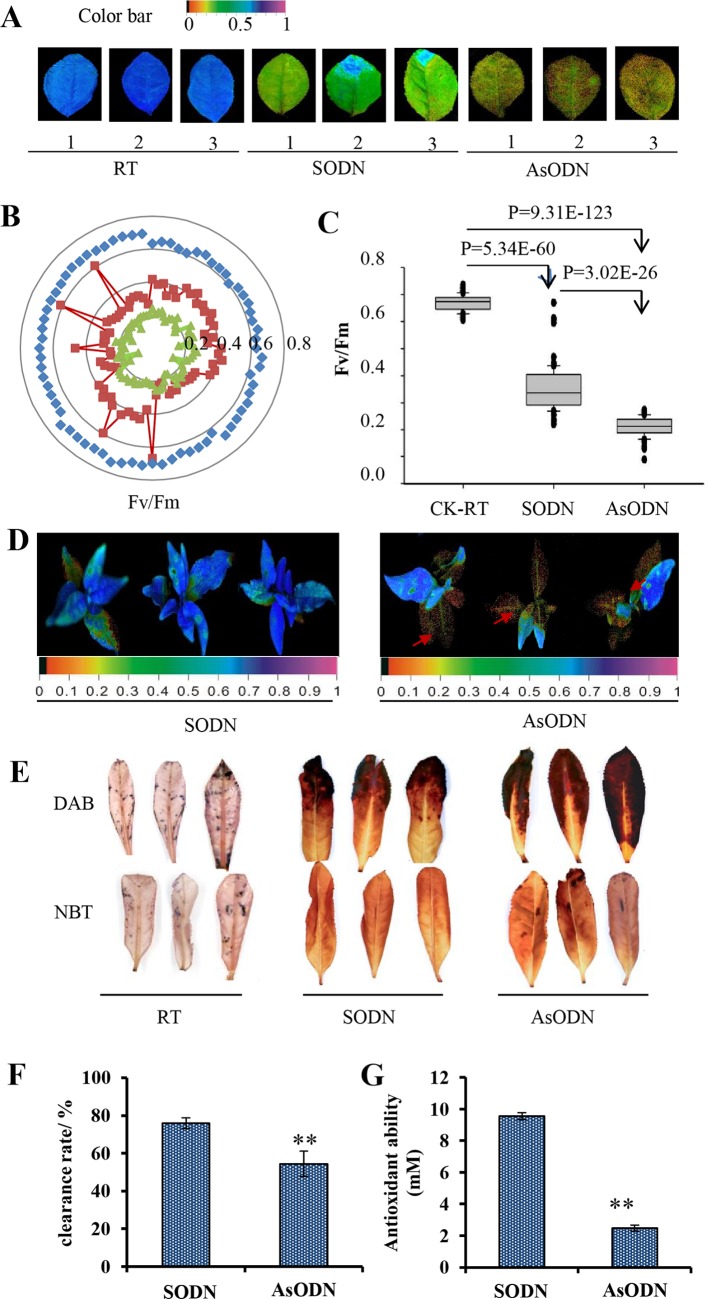
Suppression of CsUGT78A14 reduces cold tolerance in tea plant. Pseudo color image **(A)**, radar plot **(B)**, and statistical analysis **(C)** of Fv/Fm in the control and *CsUGT78A14*–silenced tea leaves under normal and cold stress conditions. Each data point presents the mean of at least three biological replicates, each of which consisted of five technical replicates. Rseudo color image in the control and CsUGT78A14–silenced tea leaves under normal and cold stress conditions **(D)**. Nitrotetrazolium blue chloride and diaminobenzidine staining of the control and *CsUGT78A14*–silenced tea leaves under normal and cold stress conditions **(E)**. Antioxidant activity analysis of flavonoids extracted from the control and *CsUGT78A14*–silenced tea leaves measured by 2,2-diphenyl-1-picrylhydrazyl **(F)** and ferric reducing ability of plasma method **(G)**.

The content of flavonol glycosides in *CsUGT78A14*-silenced tea leaves with or without a cold stress was determined and compared to study the hypothesis that *CsUGT78A14* could affect cold tolerance by glycosylation of flavonoids. As expected, the content of glycosides in *CsUGT78A14*-silenced tea plants was reduced (**Figure 4B**). LC-MS analysis confirmed that *CsUGT78A14*-silenced tea leaves produced significantly (P < 0.05) lower levels of kaempferol monoglucosides (3-glucoside and 7-glucoside) and diglucoside when compared with that in the controls (**Figure 4B**).

The concentration of total flavonols was also reduced in *CsUGT78A14-*silenced tea leaves (**Figure 4C**). To explore why the concentration of flavonoids was reduced we examined the expression levels of flavonoid synthesis-related genes *CHS*, *CHI*, *F3H*, and *FLS* in controls and *UGT78A14*-silenced tea leaves. Interestingly, the transcription of all these genes in *UGT78A14*-silenced tea plants was obviously reduced, especially under cold stress condition (**Figure 4D**). This suggested that the down-regulation of *UGT78A14* can lead to decreased expression levels of flavonoid-related genes *via* feedback inhibition of the structural genes (Yin et al., 2012), which is consistent with the observed reduction of the flavonoid content mentioned above. Therefore, *UGT78A14* appears to play a key role in modulating the formation of flavonoids and their glycosides in the tea plant.

### Suppression of *UGT78A14* Reduces Cold Tolerance in the Tea Plant

To further explore the role of *CsUGT78A14*-*1* for cold tolerance in the tea plant, Fv/Fm value was measured after to analyze the status of the photosystem II (Li et al., 2015). Purple-blue color (**Figure 4A**), together with quantitative analysis of Fv/Fm values revealed that exposure to cold stress led to the significantly damage of photosystem II (**Figures 4B**, **C**). Fv/Fm values in *CsUGT78A14*-1-silenced tea leaves (AsODN) in both cut leaves (**Figures 4B**, **C**) and leaves attached to the whole plant (**Figure 4D**) were all significantly reduced compared to the values measured for the control leaves under cold stress. The concentration of flavonoid glycosides was negatively correlated with the stress-induced damage in the tea plant, which indicated that *CsUGT78A14-1* responds to low temperatures by glycosylation of flavonoid.

### CsUGT78A14 Involved in the Regulation of Flavonoid Biosynthesis and Reactive Oxygen Species Scavenging Activity in the Tea Plant

Flavonoids can act as a potent scavenger of free radicals and superoxide radicals (Del et al., 2008). Thus, the ROS levels were determined when the expression of *CsUGT78A14* was suppressed. The control plants and *UGT78A14*-silenced tea plants were exposed to −5°C for 3 h. DAB and NBT staining was performed for detecting H_2_O_2_ and superoxide, respectively (**Figure 4E**). The *CsUGT78A14*-silenced leaves exhibited deeper and broader staining than the control leaves.

In addition, total flavonoids were extracted and subjected to FRAP and DPPH assays to test the antioxidant activity in UGT78A14-silenced plants and compared with that in the control plants (**Figures 4F**, **H**). The results showed that the *CsUGT78A14-*silenced plants have significantly lower antioxidant capacities than those of the control, suggesting that CsUGT78A14 is involved in ROS scavenging.

### CsUGT78A14 Regulates the Reactive Oxygen Species Scavenging Capacity of Flavonoid

**Figure 5:**
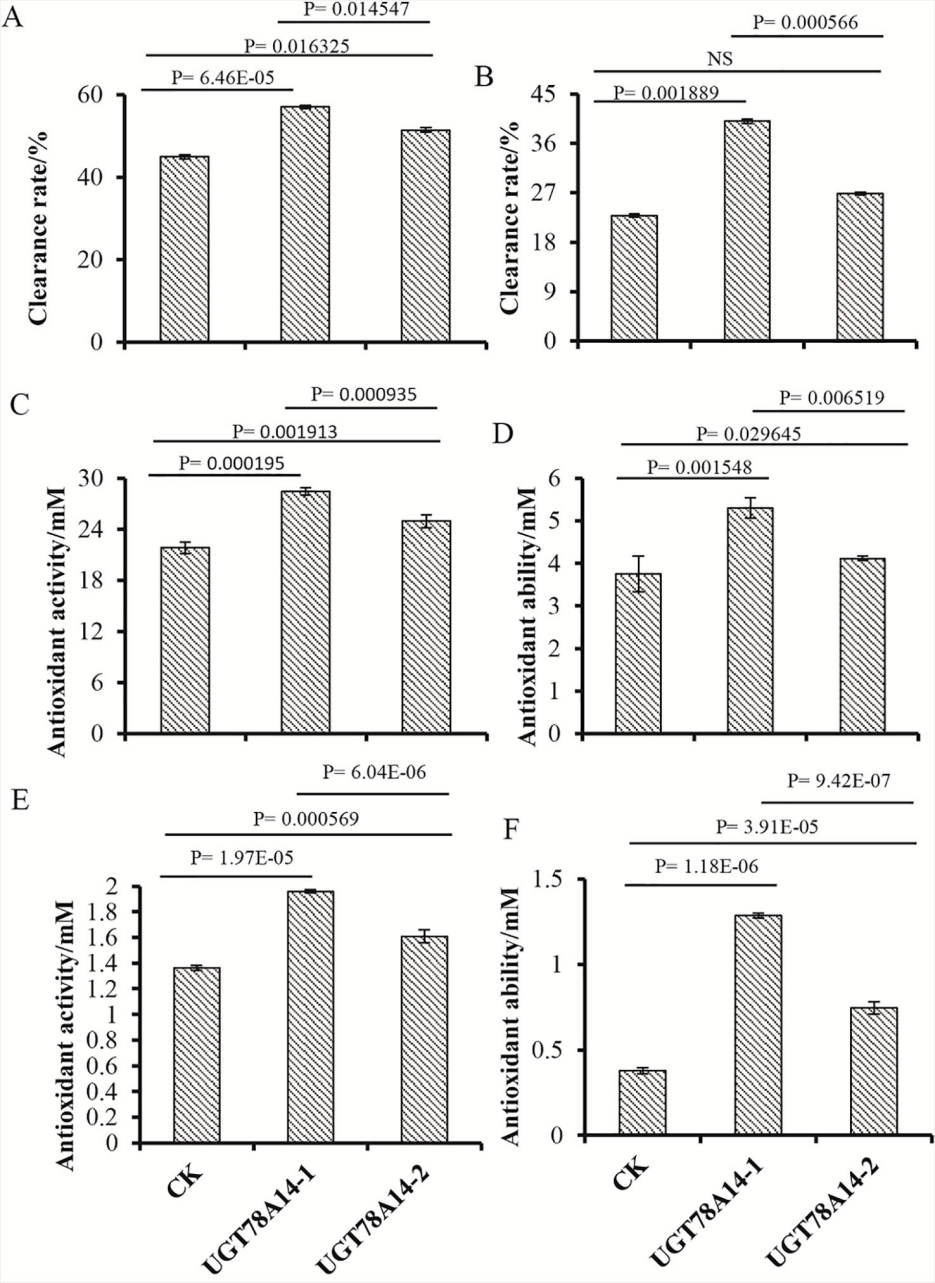
CsUGT78A14 confer flavonoids enhanced reactive oxygen species (ROS) scavenging capacity. ROS scavenging activity of reaction products formed by CsUGT78A14-1 and control was compared. Kaempferol **(A**, **C**, **E)** and quercetin **(B**, **D**, **F)** were used as substrates. ROS scavenger capacity of kaempferol and quercetin incubated with CsUGT78A14-1 was significantly enhanced compared to the control by 2,2-diphenyl-1-picrylhydrazyl **(A**, **B)**, ferric reducing ability of plasma **(C**, **D)**, and 2,2’-azino-bis(3-ethylbenzothiazoline-6-sulfonic acid) method **(E**, **F)**. Three experimental replicates were conducted for the ROS scavenging capacity analysis. NS, P=0.05141.

As kaempferol can act as a potent scavenger of free radicals and superoxide radicals (Del et al., 2008), we inferred that the glycosylation process of kaempferol probably plays a key role in response to cold stress rather than only change the accumulation of flavonoids. To test our hypothesis, we compared the antioxidant and ROS scavenging activity of glycosides and their aglycones. As the products of our enzyme is too complex, to compared the antioxidant and ROS scavenger activity of glycosides formed by the enzymes and its aglycone, here, the CsUGT78A14-1 protein and empty vector protein were incubated with kaempferol and quercetin. Both kaempferol and quercetin glycosides were formed from the reaction with CsUGT78A14-1, whereas glycosides were not present in the reaction with the control protein. The ROS scavenging activity of both reaction products from CsUGT78A14-1 and control protein was compared. The ROS scavenging capacity of kaempferol and quercetin incubated with CsUGT78A14-1 was significantly enhanced compared to the control when analyzed by the DPPH, FRAP, and ABTS method (**Figure 5**). These results demonstrate that the glucosylation process catalyzed by CsUGT78A14 significantly promoted the ROS scavenging activity of the substrates compared to their corresponding free aglycons.

## Discussion

### Products Comparison Formed by CsUGT78A14-1 and -2

Recent analysis showed that CsUGT78A14-2 is responsible for the biosynthesis of flavonol 3-O-glucoside *in vitro* (Cui et al., 2016) but its physiological role in the tea plant remained unknown. CsUGT78A14-1 differs from CsUGT78A14-2 in only one amino acid and can produce both kaempferol 3- and 7-O-glucoside.

Interestingly, CsUGT78A14-1 was also able to form kaempferol 3,7-diglucoside, which was identified by its retention index, MS and MS2 data in comparison with an authentic reference (Zhao et al., 2017). The amount of diglucoside produced by CsUGT78A14-1 was higher than that of the monoglucosides. The data indicated that Ala at 438 position of CsUGT78A14-1 plays a key role for the formation of the diglucoside *in vitro* and is located near the GSS motif, which was recently postulated as an important differentiation criterion between mono- and disaccharide-forming GTs (Huang et al., 2018; **Figure S3**).

The *in vitro* activity of UGTs can not fully reflect the *in planta* function of the enzyme, as the products formation will be affected by substrate availability (Song et al., 2015a). In this study, the *in vivo* activity of CsUGT78A14 was further studied by AsODNs-mediated silencing in the tea plant. Down-regulation of *CsUGT78A14* in the tea plant resulted in a reduced accumulation of kaempferol and quercetin monoglucosides and di-glucosides under cold stress (**Figure 3B**). Both *in vitro* and *in vivo* data confirmed that CsUGT78A14 was responsible for the biosynthesis of both flavonol monoglucosides and diglucosides in the tea plant, which have been rarely reported in plants until now.

### CsUGT78A14 Affects Cold Stress Tolerance in the Tea Plant

As cold stress affects both yield and quality of tea (Wang et al., 2017), it is urgent to develop strategies to improve cold tolerance of the tea plants (Wang et al., 2013; Yin et al., 2016). Flavonoids are a representative group of secondary metabolites and can be induced by both biotic and abiotic environmental stresses (Dixon and Paiva, 1995). Ectopic expression of *UGT76E11* enhances abiotic stress tolerance in *Arabidopsis* by increasing flavonoid accumulation (Li et al., 2018a). The *Arabidopsis* UGT79B2 and UGT79B3, identified as anthocyanin rhamnosyltransferases, contribute to cold, salt, and drought stress tolerance *via* modulating anthocyanin accumulation (Li et al., 2017). In this study, CsUGT78A14 was strongly induced in response to cold stress, promoter analysis also confirmed CsUGT78A14 contain lots of stress-responsive element (**Table S2** and **Figure S9**). The amount of flavonoid glycosides were increased when the plants were treated at cold stress and the amount of flavonoid glycosides decreased when the plants were de-acclimated (**Figure 1**). This suggested that flavonoids and their glycosides also play a key role for cold stress tolerance in the tea plant.

**Figure 6:**
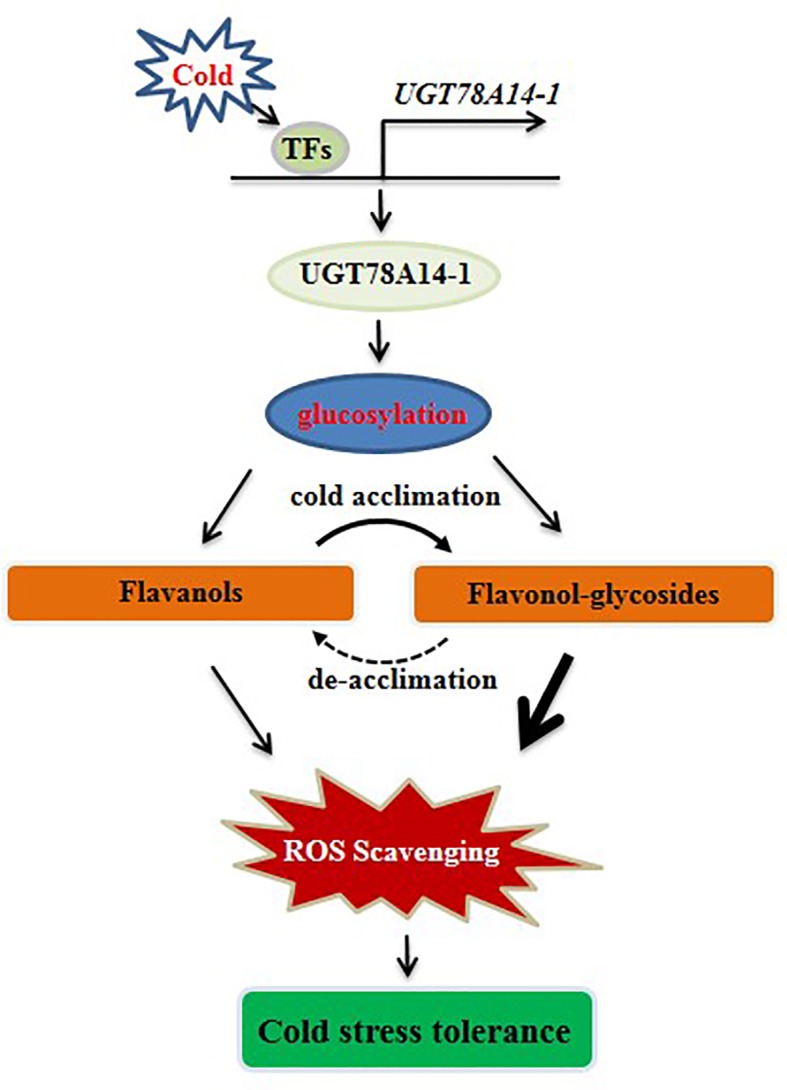
Working model of UGT78A14 involved in cold stress response in tea plant. The line thickness represents contribution ability. Different from most of the previous studies, it should be highlighted that the antioxidant capacity of flavonols aglycon was greatly enhanced by glucosylation catalyzed by CsUGT78A14-1, indicating the glycosylated products play a critical role in abiotic stresses rather than a storage forms.

Photosynthesis is sensitive to temperature fluctuations (Mondal et al., 2004; Wang et al., 2012). As an essential chlorophyll fluorescence parameter, Fv/Fm is sensitive to cold stress (Liang et al., 2007). Fv/Fm was significantly decreased in cold stress in this study (**Figure 6**), consistent with previous reports which showed that cold stress reduces Fv/Fm but promotes oxidative stress in tomato plants (Zhou et al., 2012). Fv/Fm values was significantly decreased by about 50% when exposed to cold stress (**Figures 4B**, **C**). Fv/Fm values in *CsUGT78A14*-silenced tea leaves were significantly reduced compared to the control leaves under cold stress. DAB and NBT staining showed that CsUGT78A14-silenced leaves exhibited deeper and broader staining than the wild-type leaves (**Figure 4D**). Taken together, our data showed that CsUGT78A14 plays a role in the regulation of hydrogen peroxide (H_2_O_2_) and superoxide accumulation. The reduction in Fv/Fm values might be due to the excessive accumulation of ROS under cold stress, which led to the induction of lipid peroxidation (Augspurger, 2013; Li et al., 2018b). The data presented here demonstrate that CsUGT78A14 plays a key role in the regulation of ROS scavenging capacity of flavonoids in *C. sinensis*.

### CsUGT78A14 Regulates Both Flavonols Accumulation and Reactive Oxygen Species Scavenging in Tea Plants

Plant glycosyltransferases play diverse roles in the activity modification of metabolites which might involved in the regulation of ROS homeostasis (Mittler et al., 2004). Recently, flavonols were demonstrated can act as antioxidants and reduce the production of ROS (Watkins et al., 2014). Some reports have discussed the relationship between flavonoid and abiotic stresses, but the function of flavonoids and their glycosides remained unclear.

ROS scavengers ability of flavonoids are related to the number and arrangement of their hydroxyl groups (Del et al., 2008). The hydroxyl group(s) on the aglycone is important for scavenging DPPH, and the sugar moieties do not necessary (Tohge et al., 2005a; Seyoum et al., 2006). The flavonoid aglycones have been suggested as a stronger antioxidants compared with that of their glycosides because the free hydroxyl groups play an important role in ROS scavenging (Rice-Evans et al., 1996). For instance, loss of function of UGT73B1/B2/B3, which was confirmed involved in flavonoids glycosylation, led to improvement of oxidative stress tolerance whereas UGT73B2 un-regulation increased the plants sensitivity to ROS (Chae et al., 2006). Water stress led to the accumulation of quercetin 3-O-glucosides and decreased antioxidant enzyme activities, suggested that the glycosides could act as ROS scavengers (Fini et al., 2012). Glycosyltransferases ugt73b3 and ugt73b5 mutants exhibited an accumulation of ROS in *Arabidopsis thaliana* (Simon et al., 2014). The biological role of the flavonoid glycosides is far away to be understand as they are less effective antioxidants than the corresponding aglycones (Vogt and Jones, 2000; Gachon et al., 2005) but they are accumulate during cold stress.

In the current study, the DPPH and FRAP assays showed that kaempferol glycosides exhibit a much higher antioxidant capacity compared to the control, suggesting that the modified flavonoid glycosides detected in this system have radical scavenging activities, and the glucosylation catalyzed by CsUGT78A14-1 play an important role in redox homeostasis. A very recent study showed that the antioxidative progress flavonoids can be altered by the environments, in solution quercetin glucosides showed a higher antioxidant activity than quercetin (Zheng et al., 2017), which is consistent with our current study. Therefore, the enhancement of ROS scavenging capacity of flavonoids catalyzed by CsUGT78A14 contributes at least in part the mechanism which increases cold stress resistance in the tea plant (**Figure 6**).

The antioxidant and ROS scavenging activity was further examined in *CsUGT78A14-1*-silenced tea leaves and we found that they showed a lower antioxidant capacity and reduced total flavonols compared to the control tea leaves (**Figures 4E**, **F**), consistent with the down-regulation of flavonoid synthesis-related genes *CHS*, *CHI*, *F3H*, and *FLS*. It is believed that when CsUGT78A14 was silenced, less substrates will be consumed by CsUGT78A14, which in turn inhibit the expression of the upstream enzyme genes and reduce the biosynthesis of flavonols. This result is consistent with our observation that down-regulation of CsUGT78A14 also alters the transcript levels of the upstream enzyme genes. Taken together, our results confirmed CsUGT78A14 could regulate both flavonols accumulation and ROS scavenging in response to cold stress in tea plants.

## Conclusion

CsUGT78A14 plays a critical role in cold stress by increasing flavonols accumulation and ROS scavenging capacity. These results not only enable the use of CsUGT78A14 in tea plant improvement, both to enhance cold stress tolerance and to increase flavonoid accumulation, but also provide novel insights to the underlying mechanism of the interaction of UGTs with cold stress tolerance in plant.

## Data Availability Statement

The datasets generated for this study are available on request to the corresponding author.

## Author Contributions

CS, WS, and MZ conceptualized the initial study. MZ, JJ, TG, NZ, and TJ were involved in the experimental layout. MZ, JJ, TG, TJ, JW, and QB performed the lab experiments. MZ and QB performed the RNA extraction and transcriptome analyses. CS, WS, and MZ drafted the initial article; all authors discussed the results, reviewed the article, and approved the final article.

## Funding

This work was financially supported by National Key Research and Development Program of China (2018YFD1000601), National Natural Science Foundation of China (31870678), Science Fund for Distinguished Young Scientists of Anhui Province (1908085J12). National Modern Agriculture Technology System (CARS-19), and Anhui Major Demonstration Project for Leading Talent Team on Tea Chemistry and Health.

## Conflict of Interest

The authors declare that the research was conducted in the absence of any commercial or financial relationships that could be construed as a potential conflict of interest.

## References

[B1] Augspurger C. K. (2013). Reconstructing patterns of temperature, phenology, and frost damage over 124 years: Spring damage risk is increasing. Ecology 94, 41–50. 10.1890/12-0200.1 23600239

[B2] Bolwer C. Montagu M. V. Inze D. (1992). Superoxide dismutases and stress tolerance. Annu. Rev. Plant Physiol. Plant Mol. Biol. 43, 83–116. 10.1146/annurev.arplant.43.1.83

[B3] Bowles D. Lim E.-K. Poppenberger B. Vaistij F. E. (2006). Glycosyltransferases of lipophilic small molecules. Annu. Rev. Plant Biol. 57, 567–597. 10.1146/annurev.arplant.57.032905.105429 16669774

[B4] Bradford M. M. (1976). A rapid and sensitive method for the quantitation microgram quantities of protein utilizing the principle of protein-dye binding. Anal. Biochem. 254, 248–254. 10.1006/abio.1976.9999 942051

[B5] Chae E. L. Ahn J. H. Lim J. (2006). Molecular genetic analysis of tandemly located glycosyltransferase genes, UGT73B1, UGT73B2, and UGT73B3, in Arabidopsis thaliana. J. Plant Biol. 49, 309–314. 10.1007/BF03031161

[B6] Chen L. Zhou Z. X. Yang Y. J. (2007). Genetic improvement and breeding of tea plant (Camellia sinensis) in China: from individual selection to hybridization and molecular breeding. Euphytica 154, 239–248. 10.1007/s10681-006-9292-3

[B7] Chinnusamy V. Zhu J. Zhu J. (2007). Cold stress regulation of gene expression in plants. Trends Plant Sci. 12, 444–451. 10.1016/j.tplants.2007.07.002 17855156

[B8] Cui L. Yao S. Dai X. Yin Q. Liu Y. Jiang X. (2016). Identification of UDP-glycosyltransferases involved in the biosynthesis of astringent taste compounds in tea (Camellia sinensis). J. Exp. Bot. 67, 2285–2297. 10.1093/jxb/erw053 26941235 PMC4809296

[B9] Del E. Singh R. Singh B. Singh S. Kumar N. Kumar S. (2008). Toxicology in vitro anti-free radical activities of kaempferol isolated from acacia nilotica (L.) willd. Toxicol. Vitr. 22, 1965–1970. 10.1016/j.tiv.2008.08.007 18805478

[B10] Ding Y. Lawrence C. E. (2003). A statistical sampling algorithm for RNA secondary structure prediction. Nucleic Acids Res. 31, 7280–7301. 10.1093/nar/gkg938 14654704 PMC297010

[B11] Dixon R. A. Paiva N. L. (1995). Stress-induced phenylpropanoid metabolism. Plant Cell 43, 83–116. 10.2307/3870059 PMC160915 12242399

[B12] Fini A. Guidi L. Ferrini F. Brunetti C. Di M. Biricolti S. (2012). Drought stress has contrasting effects on antioxidant enzymes activity and phenylpropanoid biosynthesis in Fraxinus ornus leaves: an excess light stress affair? J. Plant Physiol. 169, 929–939. 10.1016/j.jplph.2012.02.014 22537713

[B13] Gachon C. M. M. Langlois-Meurinne M. Saindrenan P. (2005). Plant secondary metabolism glycosyltransferases: the emerging functional analysis. Trends Plant Sci. 10, 542–549. 10.1016/j.tplants.2005.09.007 16214386

[B14] Gill S. S. Tuteja N. (2010). Reactive oxygen species and antioxidant machinery in abiotic stress tolerance in crop plants. Plant Physiol. Biochem. 48, 909–930. 10.1016/j.plaphy.2010.08.016 20870416

[B15] Hazman M. Hause B. Eiche E. Nick P. Riemann M. (2015). Increased tolerance to salt stress in OPDA-deficient rice allene oxide cyclase mutants is linked to an increased ROS-scavenging activity. J. Exp. Bot. 66, 3339–3352. 10.1093/jxb/erv142 25873666 PMC4449546

[B16] Huang F-C. Giri A. Daniilidis M. Sun G. Härtl K. Hoffmann T. (2018). Structural and functional analysis of UGT92G6 suggests evolutionary link between mono- and disaccharide glycoside forming transferases. Plant Cell Physiol. 59, 862–875. 10.1093/pcp/pcy028 29444327

[B17] Iba K. (2002). Acclimative response to temperature stress in higher plants: approaches of gene engineering for temperature tolerance. Annu. Rev. Plant Biol. 53, 225–245. 10.1146/annurev.arplant.53.100201.160729 12221974

[B18] Jing T. Zhang N. Gao T. Zhao M. Jin J. Chen Y. (2019) Glucosylation of (Z)-3-hexenol informs intraspecies interactions in plants: a case study in *Camellia sinensis*. Plant Cell Environ. 42, 1352–1367. 10.1111/pce.13479 30421786

[B19] Li X. Ahammed G. J. Zhang Y. Q. Zhang G. Q. Sun Z. H. Zhou J. (2015). Carbon dioxide enrichment alleviates heat stress by improving cellular redox homeostasis through an ABA-independent process in tomato plants. Plant Biol. 17, 81–89. 10.1111/plb.12211 24985337

[B20] Li P. Li Y. J. Zhang F. J. Zhang G. Z. Jiang X. Y. Yu H. M. (2017). The Arabidopsis UDP-glycosyltransferases UGT79B2 and UGT79B3, contribute to cold, salt and drought stress tolerance via modulating anthocyanin accumulation. Plant J. 89, 85–103. 10.1111/tpj.13324 27599367

[B21] Li Q. Yu H. M. Meng X. F. Lin J. S. Li Y. J. Hou B. K. (2018a). Ectopic expression of glycosyltransferase UGT76E11 increases flavonoid accumulation and enhances abiotic stress tolerance in Arabidopsis. Plant Biol. 20, 10–19. 10.1111/plb.12627 28902451

[B22] Li X. Ahammed G. J. Li Z. X. Zhang L. Wei J. P. Yan P. (2018b). Freezing stress deteriorates tea quality of new flush by inducing photosynthetic inhibition and oxidative stress in mature leaves. Sci. Hortic. (Amsterdam) 230, 155–160. 10.1016/j.scienta.2017.12.001

[B23] Li X. Wei J. P. Scott E. R. Liu J. W. Guo S. Li Y. (2018c). Exogenous melatonin alleviates cold stress by promoting antioxidant defense and redox homeostasis in camellia sinensis L. Molecules 23 (1), 165. 10.3390/molecules23010165 PMC6017414 29342935

[B24] Li Y. Wang X. Ban Q. Zhu X. Jiang C. Wei C. (2019). Comparative transcriptomic analysis reveals gene expression associated with cold adaptation in the tea plant *Camellia sinensis*. 20, 624 10.1186/s12864-019-5988-3 PMC6670155 31366321

[B25] Liang Y. Chen H. Tang M. Yang P. Shen S. (2007). Responses of Jatropha curcas seedlings to cold stress: photosynthesis-related proteins and chlorophyll fluorescence characteristics. Physiol. Plant 131, 508–517. 10.1111/j.1399-3054.2007.00974 18251888

[B26] Liu Z. Yan J.-P. Li D.-K. Luo Q. Yan Q. Liu Z.-B. (2015). UDP-Glucosyltransferase71C5, a major glucosyltransferase, mediates abscisic acid homeostasis in Arabidopsis. Plant Physiol. 167, 1659–1670. 10.1104/pp.15.00053 25713337 PMC4378179

[B27] Livak K. J. Schmittgen T. D. (2001). Analysis of relative gene expression data using real- time quantitative PCR and the 2(-Delta Delta C(T)). Method 408, 402–408. 10.1006/meth.2001 11846609

[B28] Mackenzie P. I. Owens I. S. Burchell B. Bock K. W. Bairoch A. Bélanger A. (1997). The UDP glycosyltransferase gene superfamily: Recommended nomenclature update based on evolutionary divergence. Pharmacogenetics 7, 255–269. 10.1097/00008571-199708000-00001 9295054

[B29] Manthey J. A. Perkins-Veazie P. (2009). Influences of harvest date and location on the levels of β -carotene, ascorbic acid, total phenols, the in vitro antioxidant capacity, and phenolic profiles of five commercial varieties of mango (Mangifera indica L.). J. Agric. Food Chem. 57, 10825–10830. 10.1021/jf902606h 19919121

[B30] Mittler R. Vanderauwera S. Gollery M. Breusegem F. V. (2004). Reactive oxygen gene network of plants. Trends Plant Sci. 9, 490–498. 10.1016/j.tplants.2004.08.009 15465684

[B31] Mondal T. K. Bhattacharya A. Laxmikumaran M. Ahuja P. S. (2004). Recent advances of tea (Camellia sinensis) biotechnology. Plant Cell 76, 195–254. 10.1023/b:ticu.0000009254.87882.71

[B32] Nakabayashi R. Yonekura-Sakakibara K. Urano K. Suzuki M. Yamada Y. Nishizawa T. (2014). Enhancement of oxidative and drought tolerance in Arabidopsis by overaccumulation of antioxidant flavonoids. Plant J. 77, 367–379. 10.1111/tpj.12388 24274116 PMC4282528

[B33] Ning J. Li X. Hicks L. M. Xiong L. (2010). A raf-like mapkkk gene dsm1 mediates drought resistance through reactive oxygen Species Scavenging in rice. Plant Physiol. 152, 876–890. 10.1104/pp.109.149856 20007444 PMC2815886

[B34] Ohgami S. Ono E. Horikawa M. Murata J. Totsuka K. Toyonaga H. (2015). Volatile glycosylation in tea plants: sequential glycosylations for the biosynthesis of aroma β -primeverosides are catalyzed by two camellia sinensis Glycosyltransferases. Plant Physiol. 168, 464–477. 10.1104/pp.15.00403 25922059 PMC4453793

[B35] Palareti G. Legnani C. Cosmi B. Antonucci E. Erba N. Poli D. (2016). Comparison between different D-Dimer cutoff values to assess the individual risk of recurrent venous thromboembolism: analysis of results obtained in the dulcis study. Int. J. Lab. Hematol. 38, 42–49. 10.1111/ijlh.12426 26362346

[B36] Rice-Evans C. A. Miller N. J. Paganga G. (1996). Structure-antioxidant activity relationships of flavonoids and phenolic acids. Free Radic. Biol. Med. 20 (7), 933–956. 10.1016/0891-5849(95)02227-9 8743980

[B37] Romero-puertas M. C. Perazzolli M. Zago E. D. Delledonne M. (2004). Microreview Nitric oxide signalling functions in plant – pathogen interactions. Cell Microbiol. 6, 795–803. 10.1111/j.1462-5822.2004.00428 15272861

[B38] Schulz E. Tohge T. Zuther E. Fernie A. R. Hincha D. K. (2016). Flavonoids are determinants of freezing tolerance and cold acclimation in Arabidopsis thaliana. Sci. Rep. 6, 1–10. 10.1038/srep34027 27658445 PMC5034326

[B39] Seyoum A. Asres K. El-Fiky F. K. (2006). Structure-radical scavenging activity relationships of flavonoids. Phytochemistry 67 (18), 2058–2070. 10.1111/j.1462-5822.2004.00428 16919302

[B40] Sheikh M. O. Halmo S. M. Patel S. Middleton D. Takeuchi H. Schafer C. M. (2017). Rapid screening of sugar-nucleotide donor specificities of putative glycosyltransferases. Glycobiology 27, 206–212. 10.1093/glycob/cww114 28177478 PMC5789813

[B41] Simon C. Langlois-Meurinne M. Didierlaurent L. Chaouch S. Bellvert F. Massoud K. (2014). The secondary metabolism glycosyltransferases UGT73B3 and UGT73B5 are components of redox status in resistance of Arabidopsis to Pseudomonas syringae pv. tomato. Plant Cell Environ. 37, 1114–1129. 10.1111/pce.12221 24131360

[B42] Song C. Gu L. Liu J. Zhao S. Hong X. Schulenburg K. (2015a). Functional characterization and substrate promiscuity of UGT71 Glycosyltransferases from Strawberry (fragaria Â ananassa). Plant Cell Physiol. 56, 2478–2493. 10.1093/pcp/pcv151 26454881

[B43] Song C. Ring L. Hoffmann T. Huang F.-C. Slovin J. P. Schwab W. (2015b). Acylphloroglucinol biosynthesis in strawberry fruit. Plant Physiol. 169, 1656–1670. 10.1007/s10681-006-9292-3 26169681 PMC4634061

[B44] Song C. Härtl K. McGraphery K. Hoffmann T. Schwab W. (2018). Attractive but toxic: emerging roles of glycosidically bound volatiles and glycosyltransferases involved in their formation. Mol. Plant 11, 1225–1236. 10.1016/j.molp.2018.09.001 30223041

[B45] Thomashow M. F. (1999). Plant cold acclimation: freezing tolerance genes and regulatory mechanisms. Annu. Rev. Plant Physiol. Plant Mol. Biol. 50, 571–599. 10.1146/annurev.arplant.50.1.571 15012220

[B46] Tognetti V. B. Van Aken O. Morreel K. Vandenbroucke K. van de Cotte B. De Clercq I. (2010). Perturbation of indole-3-butyric acid homeostasis by the udp-glucosyltransferase ugt74e2 modulates arabidopsis architecture and water stress tolerance. Plant Cell 22, 2660–2679. 10.1105/tpc.109.071316 20798329 PMC2947170

[B47] Tohge T. Matsui K. Ohme-Takagi M. Yamazaki M. Saito K. (2005a). Enhanced radical scavenging activity of genetically modified Arabidopsis seeds. Biotechnol. Lett. 27, 297–303. 10.1007/s10529-005-0683-7 15834789

[B48] Tohge T. Nishiyama Y. Hirai M. Y. Yano M. Nakajima J. I. Awazuhara M. (2005b). Functional genomics by integrated analysis of metabolome and transcriptome of Arabidopsis plants over-expressing an MYB transcription factor. Plant J. 42 (2), 218–235. 10.1111/j.1365-313X.2005.02371 15807784

[B49] Vogt T. Jones P. (2000). Glycosyltransferases in plant natural product synthesis: characterization of a supergene family. Trends Plant Sci. 5, 380–386. 10.1016/S1360-1385(00)01720-9 10973093

[B50] von Saint Paul V. Zhang W. Kanawati B. Geist B. Faus-Keßler T. Schmitt-Kopplin P. (2011). The Arabidopsis glucosyltransferase ugt76b1 conjugates isoleucic acid and modulates plant defense and senescence. Plant Cell 23, 4124–4145. 10.1105/tpc.111.088443 22080599 PMC3246326

[B51] Wang Y. Jiang C. J. Li Y. Y. Wei C. L. Deng W. W. (2012). CsICE1 and CsCBF1: two transcription factors involved in cold responses in Camellia sinensis. Plant Cell Rep. 31, 27–34. 10.1007/s00299-011-1136-5 21850593

[B52] Wang X. C. Zhao Q. Y. Ma C. L. Zhang Z. H. Cao H. L. Kong Y. M. (2013). Global transcriptome profiles of Camellia sinensis during cold acclimation. BMC Genomics 14, 415. 10.1186/471-2164-14-415 23799877 PMC3701547

[B53] Wang L. Cao H. Qian W. Yao L. Hao X. Li N. (2017). Identification of a novel bZIP transcription factor in Camellia sinensis as a negative regulator of freezing tolerance in transgenic arabidopsis. Ann. Bot. 119, 1195–1209. 10.1093/aob/mcx011 28334275 PMC5604549

[B54] Watkins J. M. Hechler P. J. Muday G. K. (2014). Ethylene-induced flavonol accumulation in guard cells suppresses reactive oxygen species and moderates stomatal aperture. Plant Physiol. 164, 1707–1717. 10.1104/pp.113.233528 24596331 PMC3982735

[B55] Winkel-Shirley B. (2002). Biosynthesis of flavonoids and effects of stress. Curr. Opin. Plant Biol. 5, 179–263. 10.1016/S1369-5266(02)00256-X 11960739

[B56] Yin R. Messner B. Faus-Kessler T. Hoffmann T. Schwab W. Hajirezaei M. R. (2012). Feedback inhibition of the general phenylpropanoid and flavonol biosynthetic pathways upon a compromised flavonol-3-O-glycosylation. J. Exp. Bot. 63, 2463–2478. 10.1093/jxb/err416 PMC3346215 22249996

[B57] Yin Y. Zhu Q. M. Z. Chen Q. C. C. (2016). Functional analysis of CsCBF3 transcription factor in tea plant (Camellia sinensis) under cold stress. Plant Growth Regul. 80, 335–343. 10.1007/s10725-016-0172-0

[B58] You J. Zong W. Hu H. Li X. Xiao J. Xiong L. (2014). A stress-responsive nac1-regulated protein phosphatase gene rice protein phosphatase18 modulates drought and oxidative stress tolerance through abscisic acid-independent reactive oxygen species scavenging in rice. Plant Physiol. 166, 2100–2114. 10.1104/pp.114.251116 25318938 PMC4256856

[B59] Zhao X. Wang P. Li M. Wang Y. Jiang X. Cui L. (2017). Functional characterization of a new tea (camellia sinensis) flavonoid glycosyltransferase. J. Agric. Food Chem. 65, 2074–2083. 10.1021/acs.jafc.6b05619 28220704

[B60] Zhao M. Zhang N. Gao T. Jin J. Jing T. Wang J. (2019). Sesquiterpene glucosylation mediated by glucosyltransferase UGT91Q2 is involved in the modulation of cold stress tolerance in tea plants. New Phytol. 10.1111/nph.16364 31828806

[B61] Zheng Y. Liao C. Zhao S. Wang C. Guo Y. (2017). The glycosyltransferase QUA1 regulates chloroplast-associated calcium signaling during salt and drought stress in *Arabidopsis*. Plant Cell Physiol. 58, 329–341. 10.1093/pcp/pcw192 28007965

[B62] Zhou J. Wang J. Shi K. Jian X. Hong Y. Quan J. (2012). Plant physiology and biochemistry hydrogen peroxide is involved in the cold acclimation-induced chilling tolerance of tomato plants ca. Plant Physiol. Biochem. 60, 141–149. 10.1016/j.plaphy.2012.07.010 22935478

