# Supplementary material for: Glucosyltransferase CsUGT78A14 Regulates Flavonols Accumulation and Reactive Oxygen Species Scavenging in Response to Cold Stress in Camellia sinensis
Source: Front Plant Sci. 2019 Dec 27;10:1675. doi: 10.3389/fpls.2019.01675 (PMC6941654; doi:10.3389/fpls.2019.01675)
Supplement: Supplementary file 1 [file DataSheet_1.pdf]

Supporting information

TITLE

Glucosyltransferase UGT78A14 Regulates Flavonols Accumulation and ROS Scavenging in Response to Cold Stress in *Camellia sinensis*

RUNNING TITLE

UGT78A14 contributes to cold stress in tea plant

AUTHOR

Mingyue Zhao[1], Jieyang Jin[1], Ting Gao[1], Na Zhang[1], Tingting Jing[1], Jingming Wang[1], Xiaochun Wan[1], Wilfried Schwab[1, 2], Chuankui Song[1]*

[1] State Key Laboratory of Tea Plant Biology and Utilization, International Joint Laboratory on Tea Chemistry and Health Effects, Anhui Agricultural University, 230036, Hefei, Anhui, P. R. China

[2] Biotechnology of Natural Products, Technische Universität München, Liesel-Beckmann-Str. 1, 85354 Freising, Germany

AUTHOR FOR CORRESPONDENCE

Chuankui Song, State Key Laboratory of Tea Plant Biology and Utilization, Anhui Agricultural University, West Changjiang Road, 230036, Hefei, Anhui, P. R. China

phone: +86-13275765280

e-mail:sckfriend@163.com

Supplementary data

TableS1. The primer sequences used in this study.

TableS2. Distribution of cis-regulatory elements scanned with promoter analysis software Plant CARE in *CsUGT78A14* gene.

FigureS1. SDS-PAGE analysis of protein extracts from E. coli expressing TEA006435, TEA001623, and two alleles of TEA007509 fusion proteins after GST-affinity purification. M, protein molecular weight marker.

FigureS2. Sequence alignment of UGT78A14-1 and UGT78A14-2 fusion proteins.

FigureS3: Enzymatic analysis of recombinant UGTs with different substrates.

FigureS4: Sugar donor performance of CsUGT78A14-1 and CsUGT78A14 -2 with kaempferol as substrate.

FigureS5. Identification of enzymatically formed products by LC-MS. Quer cetin was incubated with UDP–glucose and recombinant CsUGT78A14-1/2 and empty vector control.

FigureS6. The effect of different incubation times (0–50 min) on the product formation of CsUGT78A14 using kaempferol and UDP-glucose as substrate s. Data are presented as mean ± SE of at least three repetitions.

FigureS7. The temperature optima of CsUGT78A14. Data are presented as mean ± SE of at least three repetitions.

FigureS8. The pH optimum of CsUGT78A14. Data are presented as mean ± SE of at least three repetitions.

FigureS9. *CsUGT78A14* gene with 2000 bp fragment sequences, before ATG.

Supplementary Table S1. The primer sequences used in this study.

Purpose	Primer name	Sequence 5'-3'
Protein expression	UGT78A14-pGEX4T1-F	GGTTCCGCGTGGATCCATGAACGGTGACTCCCAACAACA
	UGT78A14-pGEX4T1-R	GGCCGCTCGAGTCGACTTAAGGGTGCTTACAAGCTTTGATTACCTCCAAC
Real Time PCR	UGT78A14-F	CCCAAGAGGAAGGGAAGAAA
	UGT78A14-R	AATAAACATCGCAACCACCA
	CHS-F	TGAAGGACCTGCCACGGTTATG
	CHS-R	GCCTTATGCTCGCTGTTTGT
	CHI-F	ATGTCTCCCTCACAGTCACCGTCC
	CHI-R	TTATTCAGCAGCAGCAGCTGTCTG
	F3H-F	ACAACAACGCTTACGGCTCTC
	F3H-R	GCCTTATGCTCGCTGTTTGT
	FLS-F	ATGGAGGTAGAGAGAGTGCAAGC
	FLS-R	TTATTGGGGAATCTTATTGAATTTG
	GADPH-F	TTGGCATCGTTGAGGGTCT
	GADPH-R	CAGTGGGAACACGGAAAGC
AsODN experiment	AsODN-1-UGT78A14	TATGCGACAGAAGCAGGTTT
	sODN-1-UGT78A14	AAACCTGCTTCTGTCGCATA
	AsODN-2-UGT78A14	TATATGCGACAGAAGCAGGT
	sODN-2-UGT78A14	ACCTGCTTGTCTCGCATATA
	AsODN-3-UGT78A14	TTCAGATCTTTCGCGGTGGC
	sODN-3-UGT78A14	CAAATGGAAGCTCAAGTGAA

Supplementary Table S2. Distribution of cis-regulatory elements scanned with promoter analysis software Plant CARE in *CsUGT78A14* gene.

Site name	Sequence	Function
AAGAA-motif	GAAAGAA	
ARE	AAACCA	cis-acting regulatory element essential for the anaerobic induction
AT~TATA-box	TATATA	
Box4	ATTAAT	part of a conserved DNA module involved in light responsiveness
BoxIII	ATCATTTTCACT	protein binding site
CAAT-box	CAAT	common cis-acting element in promoter and enhancer regions
ERE	ATTTCATA	ethylene-responsive element
G-box	TAACACGTAG	cis-acting regulatory element involved in light responsiveness
GATA-motif	AAGATAAGATT	part of a light responsive element
GCN4_motif	TGAGTCA	cis-regulatory element involved in endosperm expression
GT1-motif	GGTTAA	light responsive element
MBS	CAACTG	MYB binding site involved in drought-inducibility
MBSI	AAAAAAC(G/C)GTTA	MYB binding site involved in flavonoid biosynthetic genes regulation
MYB	CAACCA	water stress and dehydration responsiveness
MYC	CATTTG	stress responsiveness
Myb	CAACTG	water stress and dehydration responsiveness
Myc	TCTCTTA	stress responsiveness
O2-site	GATGACATGG	cis-acting regulatory element involved in zein metabolism regulation
TATA	TATAAAAT	
TATA-box	TATA	core promoter element around -30 of transcription start
TC-rich repeats	ATTCTCTAAC	cis-acting element involved in defense and stress responsiveness
TCA	TCATCTTCAT	cis-acting element involved in salicylic acid responsiveness
TCA-element	CCATCTTTTT	cis-acting element involved in salicylic acid responsiveness
TCT-motif	TCTTAC	part of a light responsive element
TGA-element	AACGAC	auxin-responsive element
Unnamed__4	CTCC	
W box	TTGACC	salicylic acid, wound and pathogen responsiveness
WRE3	CCACCT	

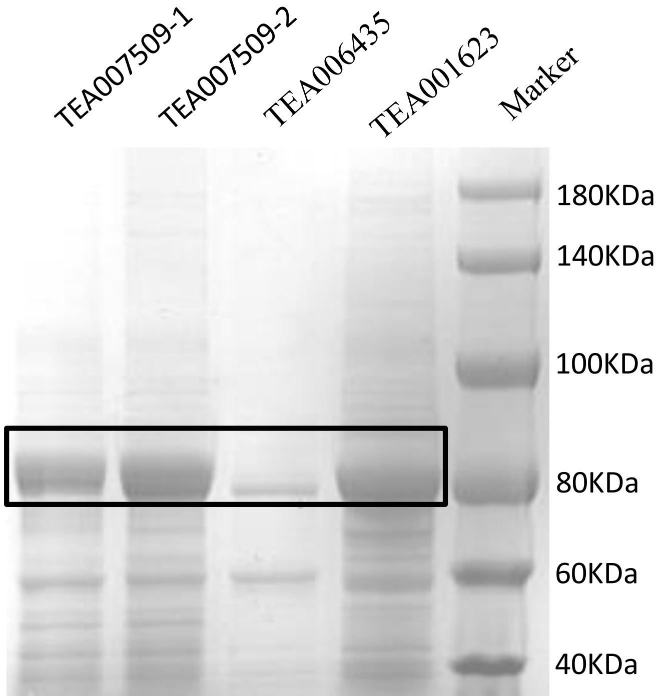

Figure S1. SDS-PAGE analysis of protein extracts from *E. coli* expressing TEA006435, TEA001623, and two alleles of TEA007509 fusion proteins after GST-affinity purification. M, protein molecular weight marker.

```
1   MNGDSQQHLHIGVLAFPFGTHAAPLLTLVRRLAAAEPTLKFSFLSTAKSNRQTFSGAKAN 60
1   ............................................................ 60
61  QFDNIKVFDVWDGVAEGDEFGGNVHVAAGLFMKATPENFKRGMEAAAAGSGVKISCLLTD 120
61  ............................................................ 120
121 AFLWFAGDLAAEMEVPWVPYWTAGSCSLSIHFYTDLIRTTLGNVNTGILENIDKTLKFIP 180
121 ............................................................ 180
181 GLSAVSVDDLPDGIILGNLESPIAQMLHNMGLKLPKAAAMVLNSFEELEPATAKDLKSKL 240
181 ............................................................ 240
241 QKVLHVGPSILSSPAPSDSDETGCLLWLDNQKPASVAYISFGTITTPPPNEVLALAETLV 300
241 ............................................................ 300
301 SSEVPFLWSLRDHSRHLFPKGFVENTVAFGKVVSWAPQLQVLAHPSVGVFVTHCGWNSIL 360
301 ............................................................ 360
361 ESITGGVPMICRPFFGDQTLNSRMVQDTWRIGVRVEGGVFTKSGTTSSLELVLSQEEGKK 420
361 ............................................................ 420
421 MRENIKVLKQNAIEAVGANGSSSENFKDLLEVIKACKHP 459
421 .................P..................... 459
```

Figure S2. Sequence alignment of UGT78A14-1 and UGT78A14-2 fusion proteins

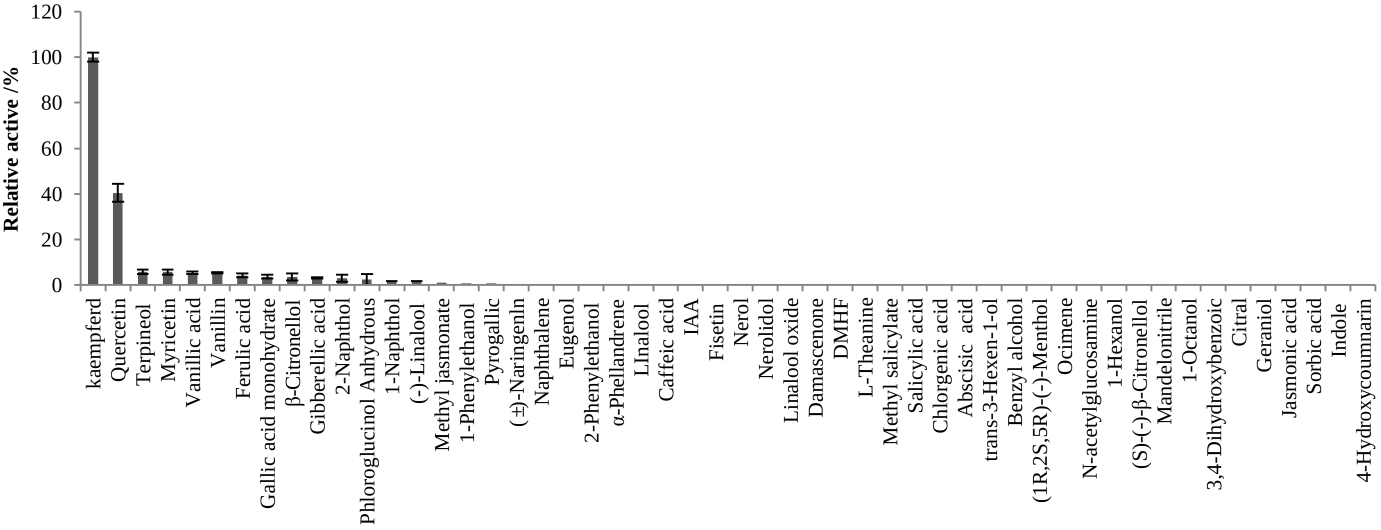

Figure S3: Enzymatic analysis of recombinant UGT proteins with different substrates. Relative specific activity of CsUGT78A14 proteins from *Camellia sinensis* towards putative substrates as determined by UDP Glo-glycosyltransferase assay kit. Data for three technical replicates are shown.

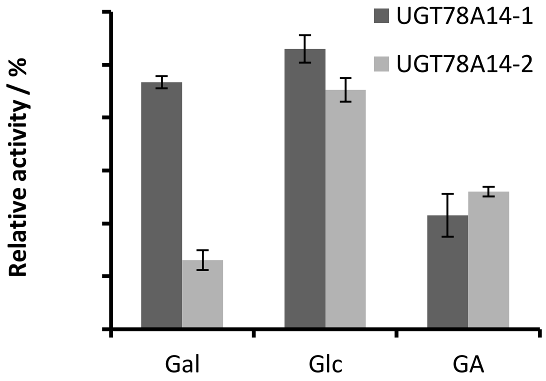

Figure S4: Sugar donor performance of CsUGT78A14-1 and CsUGT78A14-2 with kaempferol as substrate.

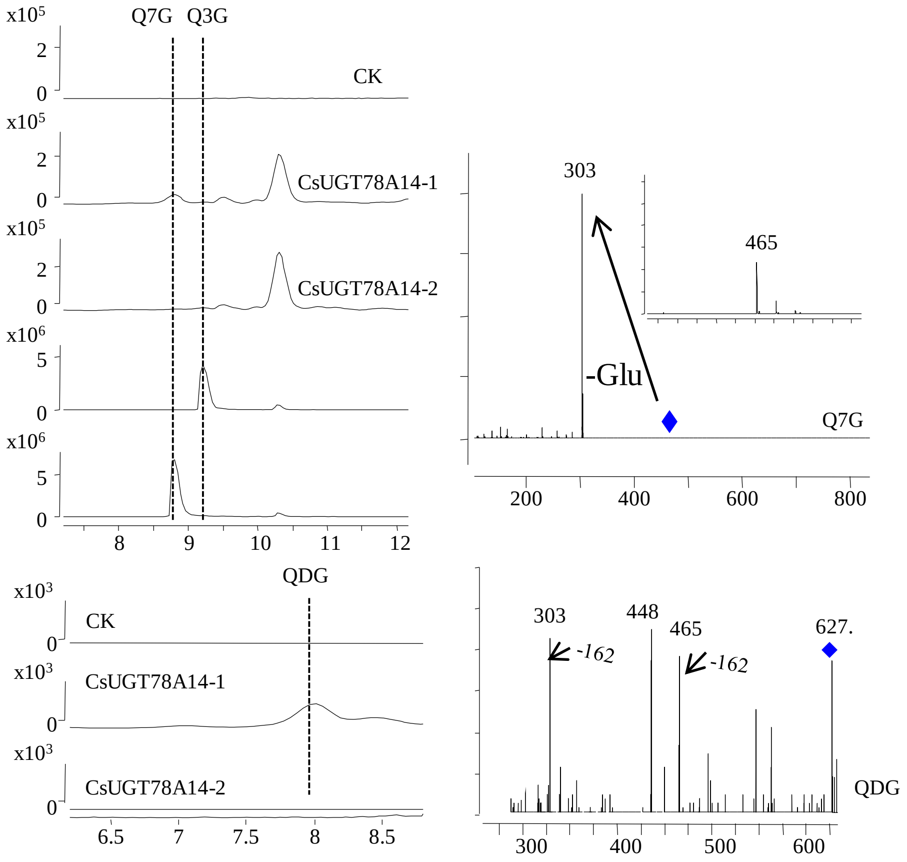

Figure S5. Identification of enzymatically formed products by LC-MS. Quercetin was incubated with UDP–glucose and recombinant CsUGT78A14-1/2 and empty vector control.

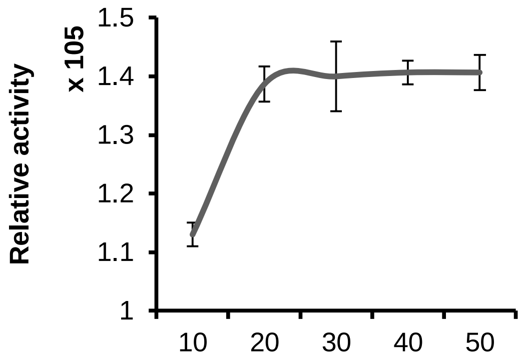

Figure S6. The effect of different incubation times (0–50 min) on the product formation of CsUGT78A14 using kaempferol and UDP-glucose as substrates. Data are presented as mean ± SE of at least three repetitions.

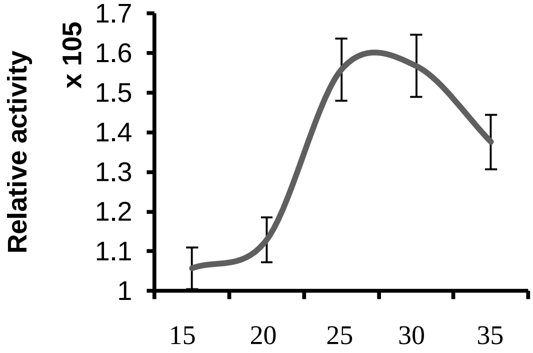

Figure S7. The temperature optima of CsUGT78A14. Data are presented as mean ± SE of at least three repetitions.

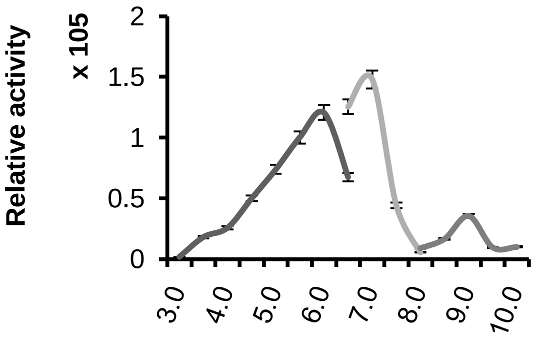

Figure S8. The pH optimum of CsUGT78A14. Data are presented as mean ± SE of at least three repetitions.

```
+ AATACGAACT CTGATAAATA AAAGTACATT ATTTCTATCA ACATTCTGTT TTAAGGATTG AATTTATTGT
- TTATGCTTGA GACTATTTAT TTTCATGTAA TAAAGATAGT TGTAAGACAA AATTCCTAAC TTAAATAACA

+ AATAATTTGG GGGTGTTACA TAGGCTGAGA TTGTTGTCGG AGATGGCAAT AGCAGTTGAG GAGATGGGGC
- TTATTAAACC CCCACAATGT ATCCGACTCT AACAACAGCC TCTACCGTTA TCGTCAACTC CTCTACCCCG

+ GATTAGAAAG ACTATAAGTA TAATTCGTTC TTGTTTGTGA GCTCAACTTT TTGATAATTA ATTATTTTTC
- CTAATCTTTC TGATATTCAT ATTAAGCAAG AACAAACACT CGAGTTGAAA AACTATTAAT TAATAAAAAG

+ ACTAAGGAAA ATCGAAACAC ACAGTACAAA TAGTTTATTT GTTGCAACTT ACTATAAGTT AAAGTTGATA
- TGATTCCTTT TAGCTTTGTG TGTCATGTTT ATCAAATAAA CAACGTTGAA TGATATTCAA TTTCAACTAT

+ ATCAAAAGTG TACATAAAAT AAAAATAAAA ATAAAAACAC TCCTCTCATA ACATGTAGAT GATCAATTTT
- TAGTTTTCAC ATGTATTTTA TTTTTATTTT TATTTTTGTG AGGAGAGTAT TGTACATCTA CTAGTTAAAA

+ TTTTGATTTA TTTAAATTTC ATCTAACAAA CTATAAGTAA AAAAAAATTA TATTAAACAA CCTCATGCAA
- AAAACTAAAT AAATTTAAAG TAGATTGTTT GATATTCATT TTTTTTTAAT ATAATTTGTT GGAGTACGTT

+ GCTCGCAAAT AAAATAGAGT ATAGACCCTC AAAATGGTGT ATACGCAAGT TAAGTTATAT GCCAGCTTGA
- CGAGCGTTTA TTTTATCTCA TATCTGGGAG TTTTACCACA TATGCGTTCA ATTCAATATA CGGTCGAACT

+ TTAACACAAA TGACATAATT CAATATTATG AGTCATAATT AAAAAAAATA TTGAGTTATA GCAAAATATC
- AATTGTGTTT ACTGTATTAA GTTATAATAC TCAGTATTAA TTTTTTTTAT AACTCAATAT CGTTTTATAG

+ ACATAAGATA TATAATTAAA CAATTCTTGA ATCATAATCA AAATAACATC ATAATCACTA TCACAATATG
- TGTATTCTAT ATATTAATTT GTTAAGAACT TAGTATTAGT TTTATTGTAG TATTAGTGAT AGTGTTATAC

+ TCATATAAAT AACATGCTAA TTTAACTTGG GAAGTGGGTT AAAAGAGATG ACATTATAAA ATTTATTTAA
- AGTATATTTA TTGTACGATT AAATTGAACC CTTCACCCAA TTTTCTCTAC TGTAATATTT TAAATAAATT

+ TTTTTATTTA ATAGAAAAAT ATTAAAGAGA TGACATTGCT TATAAATGAT GTGCTTTAAA TAAAAATTAT
- AAAAATAAAT TATCTTTTTA TAATTTCTCT ACTGTAACGA ATATTTACTA CACGAAATTT ATTTTTAATA

+ TAAACAGTCT TAGAATATCA TATAGCATTG CCTAGTGATA CTCAAAACAT AAGAAATCAC AAATTACATT
- ATTTGTCAGA ATCTTATAGT ATATCGTAAC GGATCACTAT GAGTTTTGTA TTCTTTAGTG TTTAATGTAA

+ AAGTAGTCTG TCAACTGCCT GAAAATAATG AGTTTATTAT ATCCAATTTA TAAGATTTTT GACTATATTA
- TTCATCAGAC AGTTGACGGA CTTTTATTAC TCAAATAATA TAGGTTAAAT ATTCTAAAAA CTGATATAAT

+ TTGATTTAAA CTACTACATA AATAATATAA TATAGTACTC TTAAAGTATC AAATAACTAT TTATCGTGCA
- AACTAAATTT GATGATGTAT TTATTATATT ATATCATGAG AATTTCATAG TTTATTGATA AATAGCACGT

+ CATGAACCCT GGGCCTTCTT TTTTTCTGTC ACTGTCTGTC AGATTATTTG ATATAATTTC TATCCATAAT
- GTACTTGGGA CCCGGAAGAA AAAAAGACAG TGACAGACAG TCTAATAAAC TATATTAAAG ATAGGTATTA

+ TTATTACCTA AAAACAATTT AGATAAACAG AAAAAAAACT CATTATTTAA AACTATAGAA GGAGTAGCTT
- AATAATGGAT TTTTGTTAAA TCTATTTGTC TTTTTTTTGA GTAATAAATT TTGATATCTT CCTCATCGAA

+ TTGCTTGCTA GTTACTACTT TTAACTTAAA AAAAAAAAAA CCTTTACCAA ACAGGTTATT GTCCTGCACT
- AACGAACGAT CAATGATGAA AATTGAATTT TTTTTTTTTT GGAAATGGTT TGTCCAATAA CAGGACGTGA

+ TCGTTTTGGA CTTTAGGAGG TGGGTTAATT TTTTAATTTT GAAACTTTTT AAATATTTAA GGTCAAATTT
- AGCAAAACCT GAAATCCTCC ACCCAATTAA AAAATTAAAA CTTTGAAAAA TTTATAAATT CCAGTTTAAA

+ GGCATGATTG AAAAAGACA AAAAGTTAAA AAGTCACTAT CCTATTTAAG GGCTAGTTTG GCATGTACTA
- CCGTACTAAC TTTTTTCTGT TTTTCAATTT TTCAGTGATA GGATAAATTC CCGATCAAAC CGTACATGAT

+ AATAAGCCAA AAAGTTAAAA AGTCACTATC TTATTTAAGT CACATCATCT TATTTAAATC ACATCACCTC
- TTATTCGGTT TTTCAATTTT TCAGTGATAG AATAAATTCA GTGTAGTAGA ATAAATTTAG TGTAGTGGAG

+ TCTAACACAT CACATCAAAC CCAACCCAAA CCCAACCCAA ACCCACCAAT AAGTCAAAAA GTGCACTTTT
- AGATTGTGTA GTGTAGTTTG GGTTGGGTTT GGGTTGGGTT TGGGTGGTTA TTCAGTTTTT CACGTGAAAA

+ TTAGGACATC CCAAACCAAC CATAAGTCAC ATCATCTTAT TTAAGTCACA TTACCTCTCT AACACATCAC
- AATCCTGTAG GGTTTGGTTG GTATTCAGTG TAGTAGAATA AATTCAGTGT AATGGAGAGA TTGTGTAGTG

+ TTCAAACCCA ATCCAAACCC AACCCATATT TCATAAAAAG TCAAAAAGTA CACTTATTTA GTCTTCCCAA
- AAGTTTGGGT TAGGTTTGGG TTGGGTATAA AGTATTTTTC AGTTTTTCAT GTGAATAAAT CAGAAGGGTT

+ ACTAGGCCTA AATTTGGAGT TAAAAAAGTT TCACTTAGCA TATATTTTTT TGATTCGGTT CCGTTACGTA
- TGATCCGGAT TTAAACCTCA ATTTTTTCAA AGTGAATCGT ATATAAAAAA ACTAAGCCAA GGCAATGCAT

+ GCTCGAAATA TCAAAGGCTC GAAATCTCAA AACGACGTCG TTTTAAGGGT TTTTTTTTTA TTCTCTTACC
- CGAGCTTTAT AGTTTCCGAG CTTTAGAGTT TTGCTGCAGC AAAATTCCCA AAAAAAAAAT AAGAGAATGG

+ GGTAAGATAA TATTTTCCGT ACCATTCGAG TCATTCGAGC TCTACACATT CGAGCTACGG ATCATTTTCA
- CCATTCTATT ATAAAAGGCA TGGTAAGCTC AGTAAGCTCG AGATGTGTAA GCTCGATGCC TAGTAAAAGT

+ TTTTTGATTT TTTTAATTTT TATGAAAGAA AGAAATAAAA TAATTATAAT TTAAATATAA TATTTTATCT
- AAAAACTAAA AAAATTAAAA ATACTTTCTT TCTTTATTTT ATTAATATTA AATTTATATT ATAAAATAGA

+ TATTAATATT TTAATTTATT TTATACCTAA AAAATCCAAA AAATCTATAT CAAATGAAGT TTATATATTG
- ATAATTATAA AATTAAATAA AATATGGATT TTTTAGGTTT TTTAGATATA GTTTACTTCA AATATATAAC

+ TCCTGTTCAC TCCAAACTAG TGATCCCACA AAACCAAC
- AGGACAAGTG AGGTTTGATC ACTAGGGTGT TTTGGTTG
```

- AAGAA-motif
- ARE
- AT~TATA-box
- Box 4
- Box III
- CAAT-box
- ERE
- G-box
- GATA-motif
- GCN4_motif
- GT1-motif
- MBS
- MBSI
- MYB
- MYC
- Myb
- Myc
- O2-site
- TATA
- TATA-box
- TC-rich repeats
- TCA
- TCA-element
- TCT-motif
- TGA-element
- Unnamed__4
- W box
- WRE3

Figure S9. *CsUGT78A14* gene with 2000 bp fragment sequences, before ATG.
